# 3D-Printed Gastric Resident Electronics

**DOI:** 10.1002/admt.201800490

**Published:** 2018-12-13

**Authors:** Yong Lin Kong, Xingyu Zou, Caitlin A. McCandler, Ameya R. Kirtane, Shen Ning, Jianlin Zhou, Abubakar Abid, Mousa Jafari, Jaimie Rogner, Daniel Minahan, Joy E. Collins, Shane McDonnell, Cody Cleveland, Taylor Bensel, Siid Tamang, Graham Arrick, Alla Gimbel, Tiffany Hua, Udayan Ghosh, Vance Soares, Nancy Wang, Aniket Wahane, Alison Hayward, Shiyi Zhang, Brian R. Smith, Robert Langer, Giovanni Traverso

**Affiliations:** 1 Department of Mechanical Engineering University of Utah Salt Lake City, UT 84112, USA; 2 Boston University School of Medicine 72 E Concord St, Boston, MA 02118, USA; 3 Charles Stark Draper Laboratory Cambridge, MA 02139, USA; 4 Institute for Medical Engineering and Science Massachusetts Institute of Technology Cambridge, MA 02139, USA; 5 Division of Gastroenterology Brigham and Women’s Hospital Harvard Medical School Boston, MA 02115, USA; 6 Department of Mechanical Engineering Massachusetts Institute of Technology Cambridge, MA 02139, USA

**Keywords:** 3D printing, biomedical devices, gastric resident devices, gastric resident electronics, ingestible electronics

## Abstract

Long-term implantation of biomedical electronics into the human body enables advanced diagnostic and therapeutic functionalities. However, most long-term resident electronics devices require invasive procedures for implantation as well as a specialized receiver for communication. Here, a gastric resident electronic (GRE) system that leverages the anatomical space offered by the gastric environment to enable residence of an orally delivered platform of such devices within the human body is presented. The GRE is capable of directly interfacing with portable consumer personal electronics through Bluetooth, a widely adopted wireless protocol. In contrast to the passive day-long gastric residence achieved with prior ingestible electronics, advancement in multimaterial prototyping enables the GRE to reside in the hostile gastric environment for a maximum of 36 d and maintain ≈15 d of wireless electronics communications as evidenced by the studies in a porcine model. Indeed, the synergistic integration of reconfigurable gastric-residence structure, drug release modules, and wireless electronics could ultimately enable the next-generation remote diagnostic and automated therapeutic strategies.

The integration of electronics with the human body has the potential for significant impact on novel personalized diagnostic and treatment strategies. For instance, the creation of wearable electronics^[1]^ has enabled real-time interfacing of digital devices with the body to measure physiological parameters such as heart rate, respiratory, oxygen saturation, blood pressure, and glucose level.^[2]^ Implantable electronics have enabled a broad set of capabilities including electrical stimulation of several organs including the heart,^[3]^ the gastrointestinal tract,^[4]^ and the brain,^[5]^ as well as monitoring of physiologic parameters including cardiac^[6]^ and gastrointestinal.^[7]^ Moreover, several technologies including systems allowing externally controllable drug release in the form of microchips as well as pump systems are in various stages of development.^[8–10]^ All these systems require a range of significant intervention ranging from needle-based access to surgical implantation. Furthermore, long-term surgically placed medical implants are associated with eliciting foreign body immune responses.^[9,11]^ In addition, implanted devices can serve as a nidus for infection which can require immediate operative intervention.^[12]^

Oral delivery remains the preferred route for drug delivery and is an intuitive, appealing but relatively unexplored method of transiently implanting long-term resident electronics. Oral delivery can leverage the significant space and immune-tolerant environment available within the gastrointestinal tract, circumventing the needs for more invasive device placement. This method, coupled with a unique design, optimized set of materials, and the capacity to control the macrostructure may obviate the potential health risks often associated with surgical implantation. As noted above, the stomach represents an immune privileged site in the body with a holding volume of ≈1.5 L^[13]^ without significant distention. The stomach is an organ that has evolved to digest a large volume of food and as such it has a relatively higher tolerance for foreign materials. Pathophysiologic examples of the tolerance of the stomach for resident objects have been documented for centuries in the form of bezoars.^[14]^ These aggregates can form from many materials^[15]^ and generally manifest in gastrointestinal outlet obstruction symptoms when they reach a mass in excess of ≈50 g.^[16]^ Long-term (>1 week) larger devices have been applied successfully to the stomach for bariatric intervention.^[17]^ Gastric resident systems in ingestible formats are in various stages of preclinical and clinical development and are being applied for drug delivery supporting the capacity of this environment to sustain a range of materials and even drugs for prolonged periods of time.^[18–20]^

The delivery of electronics through ingestion is an exciting concept that has been explored since 1957.^[21]^ Recent developments in ingestibles^[22]^ have noted a myriad of functionalities, incorporating temperature,^[23]^ pH,^[24]^ pressure,^[25]^ or biomolecular^[26]^ sensors, wireless identification microchip,^[27]^ gas sensor,^[28]^ camera for wireless imaging and endoscopy,^[29]^ or drug delivery modules.^[30]^ Nevertheless, these ingestible electronics are incapable of maintaining a stable long-residence in the stomach. Most demonstrations to date are limited to a passive, uncontrolled gastric residence with a period of less than a week, which limits the potential application of ingestible bioelectronics to transient diagnostics and therapeutic strategies.

Here, we demonstrate the design and manufacturing of a wireless gastric resident electronic (GRE) device that can achieve in vivo gastric residence in a porcine stomach for up to a maximum of 36 d and maintaining in vivo wireless communication for a maximum of 15.3 d. We synthesized controlled-release formulation of drugs (antimicrobial and hormonal agents) that can be cointegrated in the drug delivery module to enable the simultaneous controlled-release of drugs. A customized multimaterial 3D printing of gastric residence architecture allows a seamless integration of wireless electronics, transformable multimaterials structure, and drug delivery reservoirs, as shown in Figure 1A. The GRE is designed to be delivered orally into the stomach (1), reside in the stomach (2), pass through the pylorus (3), and be excreted out of the body. The device can be folded into an ingestible dosage form for delivery via the oral tract, as described in Figure 1C. Upon reaching the stomach, the system expands to a geometry with an effective diameter that is larger than the pylorus (approximate diameter of 1.9 cm^[31]^) to enable the residence of the device in the gastric space as shown in Figure 1D. This coupled to the mechanical properties of the central flexible element (see Figure S1, Supporting Information) enables gastric residence and allows long-term remote communication with a personal device.

**Figure 1:**
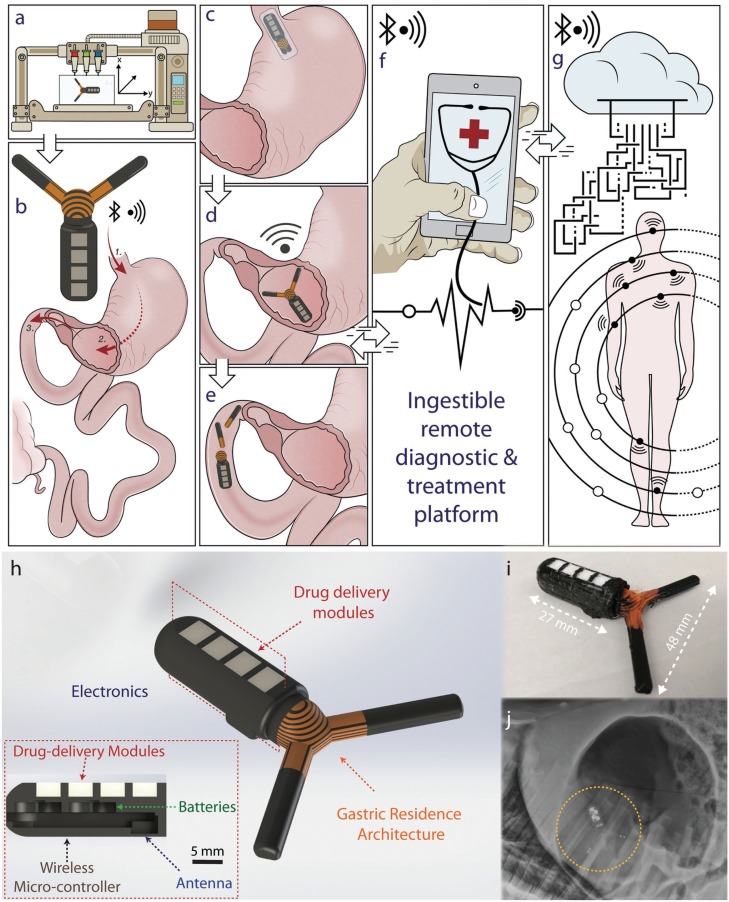
3D-printed gastric resident electronics (GRE) for biomedical applications. Illustration describes the 3D-printed GRE concept: A) patient-specific multimaterial 3D printing of GRE. B) GRE is designed to be delivered orally (1), reside in the stomach for weeks (2), and finally break up (3) pass through the pylorus and be excreted from the gastric space. C) Specifically, the GRE can be compressed into a capsule-size dosage form. D) The expansion of the device enables gastric residence and allows long-term remote communication with personal device. E) Ultimately, the disintegration of the device allows the safe passage of the device from the gastric space. F) GRE is directly compatible with personal devices, such as a smart phone, empowering the users to communicate and control the long-residence device without a specialized equipment. G) This enables a seamless interconnection with other wireless electronics peripherals, wearable devices, and biomedical implants, allowing a real-time feedback-based automated treatment or responsive medication. The interconnection of GRE with the digital cloud via personal electronics could ultimately enable the next generation of digital medical interventions. H) Computer-aided design models of the gastric-resident electronics device showing the (i) gastric resident architecture; (ii) integration of electronics and power system for communications and control; (iii) personalized drug delivery modules. Inset shows the cross section of the design demonstrating the integration of a Bluetooth wireless-microcontroller, antenna, batteries, and drug delivery modules. I) Optical photograph shows the dimension of a fabricated device. J) X-ray image shows the deployed GRE in a porcine stomach.

Ultimately, the passive disintegration of the device or potential triggered disintegration allows the passage of the device from the gastric cavity as illustrated in Figure 1E. The extended residence property of the gastric electronics can potentially help realize the next generation of digital diagnosis and treatment strategies. For instance, as described in Figure 1F, GRE can be compatible with personal electronics, such as the smart phone, enabling the users and health care providers to directly communicate and control the GRE through Bluetooth connection without specialized equipment. This compatibility also allows a seamless interconnection with other wireless electronic peripherals, wearable devices, and biomedical implants, facilitating a real-time feedback-based automated treatment or responsive medication. Further, as illustrated in Figure 1G, the interconnection of GRE with the digital cloud via personal electronics could ultimately enable remote health management and monitoring^[32]^ as well as personalized^[33]^ and large population data collections for clinical studies.

Several fundamental challenges had to be overcome before realizing the GRE. First, the device has to transform from an ingestible dosage form to an expanded configuration immediately upon the entry into the stomach. Second, the GRE must maintain its capacity for residing in the stomach within the mechanically and chemically hostile gastric environment and have the capacity to be triggered to break up into subcomponents to enable gastrointestinal transit in the event of an adverse reaction. Third, GRE must be compatible with a widely adopted wireless communications protocol (e.g., Bluetooth) and be capable of maintaining long-term (beyond a day) communications with personal electronics devices. Indeed, such level of integration has not been demonstrated with prior ingestible devices, partly due to the limitation in the versatility of design and integration of conventional manufacturing method such as molding.

Here, we overcome these challenges with a unique 3D, heterogeneous design enabled by multimaterials additive manufacturing.^[34]^ Specifically, we develop a wireless remotely controlled drug-release module integrated with a two-armed gastric residence architecture that can transform between a compressed dosage form to an expanded form (Figure 1H). The robustness of the device is tailored to achieve a prescribed gastric residence with the dynamic gastric environment. This hybrid integration approach leverages the versatility of additive manufacturing design methodology, and enables the seamless incorporation of gastric residence architecture with active modules such as personalized drug delivery modules, wireless electronics, antenna, and power systems to achieve a long-term in vivo communications and drug delivery.

Prior successful gastric resident architectures generally rely on synthesized materials with a similar chemical basis^[19]^ to maintain strong interfacial strength between the elastomeric and stiff component. Here, we applied the layer-by-layer 3D printing of elastomeric and stiff polymer to significantly amplify the adhesion strength between the two different classes of materials that would otherwise be fragile in a dynamically hostile gastric environment. In order to accommodate commercially available wireless electronic components, we developed a three-armed based system for gastric residency. We first investigated the freeform fabrication of a robust transformable architecture prototype with fused deposition modeling (FDM) based on commercially available thermoplastic filaments (see Figure 2A). Specifically, the gastric resident architecture is created with a biocompatible poly-l-lactic acid (PLA) and a thermoplastic polyurethane (NinjaTek NinjaFlex 85A). The gastric-residence architecture prototype is designed with the maximum compressed thickness that fits into a 000 size capsule (hereafter referred to as GRA).

**Figure 2:**
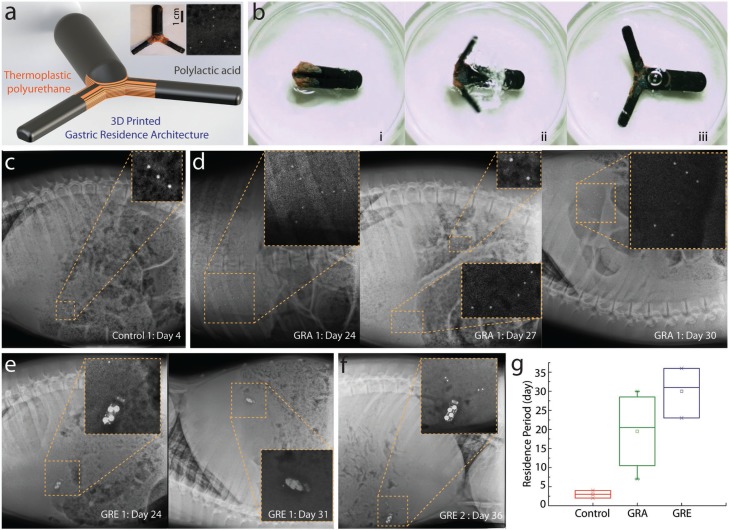
3D-printed multimaterial gastric-residence architecture prototype (GRA) and electronics (GRE). A) Schematic of the computer-aided design model of the 3D-printed multimaterial architecture. Left inset image shows the optical photograph of a 3D-printed multimaterial GRA and right inset is an X-ray image indicating the relative location of metal probes embedded in the GRA of the in vivo gastric residence study. B) High-speed camera imaging series showing the expansion of 3D-printed architecture (i) before, (ii) during, and (iii) after expansion. C) X-ray image shows the gastric residence of a control prototype demonstrating the maximum gastric residence of 4 d without a gastric residence architecture. D) In contrast, GRA permits gastric residence for up to 24 d, as shown in the X-ray image. The structure will subsequently disintegrate by detachment. First, one of the GRA arm is detached, as indicated by the metal probes at day 27. (The top inset image shows the detached arm that has been passed to the intestinal region, while the remaining structure stays in the gastric space.) Second, at day 30, both GRA arms are detached, allowing the GRA to pass between day 31 and day 33. E) GRE exhibited a similar disintegration as GRA where at day 24, one of the prototypes began to lose one of the gastric residence structure, before both gastric residence arms are detached. F) In another GRE, a maximum gastric residence of 36 d was achieved. G) Statistical comparison of device residence period of a structure without GRA (control), GRA prototype, and ultimately GRE, demonstrating the effectiveness of GRA in prolonging gastric residence.

The reconfigurable structure (i.e., the GRA) allows the transformation from an ingestible form to an expanded form in the stomach. The structure can be folded into a gelatin capsule that can dissolve in the gastric fluid. Upon the dissolution, it expands to an effective diameter that is larger than the diameter of the pylorus, which coupled to the mechanical properties of the multi-layered flexible center, enables gastric residency (see Figure S1, Supporting Information). Figure 2B, shows the expansion of the device upon full immersion in simulated gastric fluid, independent of the orientation of the device. We note that the expansion occurs within 50 s upon full immersion. Next, the GRA was embedded with stainless steel imaging probes (1 mm beads) to enable X-ray visualization of the printed device inside the gastric cavity (see inset of Figure 2A). Specifically, we inserted three metal fiducials to indicate the location of the potential electronics chipset (hereafter referred to as head), and two metal fiducials to indicate the two thinner arms supporting gastric residence architectures (hereafter referred to as arm). This allowed for the measurement of the gastric residence period by monitoring the potential electronics site (head) as well as the structural integrity of the printed multimaterial prototype without an endoscopy procedure. We defined the gastric residence period as the maximum time the head is detected on X-ray. We noted that there is an imaging gap in some of the studies due to the limitation of maximum possible X-ray frequency under the approved animal protocol.

Control experiments were conducted through evaluation of the gastric residence period of the electronics head without the gastric residence architecture. This is a critical consideration to account for the slower gastric motility of a porcine model.^[35]^ We noted that the maximum gastric residence period achieved without GRA is 3 d, as shown in Figure 2C. In contrast, the GRA significantly prolonged the gastric residence. The GRA remained intact after 24 d in the gastric environment, as shown in Figure 2D (left). Next, we evaluated the disintegration of the prototype. In two of the four samples, we were able to capture the images of the failure that shows the prototype detachment of arms, which eventually causes the passage of the GRA from the gastric space. As shown in Figure 2D (center), the GRA structure disintegrated via the initial detachment of one of the two arms, as indicated by the metal probes at day 27. On day 30 (Figure 2D right), both the GRA arms had been detached, which ultimately caused the GRA to pass from the gastric space within 34 d. In one of the four studies, a premature passage of the GRA was observed on day 7 without disintegration. One of the four samples’ failure mechanism was not captured due to the imaging frequency limited by the animal protocol. Nevertheless, no clinical complication (such as intestinal obstruction) was observed in the experiments, indicating that GRA was passed safely. This can potentially be attributed to the fenestrated open macrostructure of the GRA that reduces the likelihood of intestinal blockages, in contrast to prior clinical complications observed with closed macrostructures of polyurethane foams.^[36]^ Future studies with a larger number of samples can further evaluate the clinical safety of the prototype. We have also demonstrated an approach to incorporate a thermoset polymer by first coprinting with water-soluble polyvinyl alcohol (PVA) polymer, and subsequently replacing the PVA with the thermoset elastomeric polymer. As a proof of concept, we have created a PCL-PLA-based GRA, which demonstrates the potential for future work to incorporate a thermoset polymer (see Figure S2, Supporting Information). Future work can also incorporate a pH-responsive enteric elastomer,^[37]^ which can dissolve in the neutral-pH environment of the small and large intestine. Additionally, we developed prototypic modes of external triggering of dissolution of linker segments in the event that macrostructure dissolution would be required should a subject develop an adverse reaction to a device (see Figure S3, Supporting Information).

Based on the in vivo experiments conducted with the GRA, we increased to the dimension indicated in Figure 1I. We note that the minimum size of the head of the GRE is larger in comparison to the GRA to accommodate the minimum size of the electronics chipset design possible without circuit board components integration. Indeed, the electronic prototype board can be readily miniaturized with a chipset repackaging in the future work; we note that this integration is beyond the scope of this project as the cost of such integration per device is significant for a small number of prototypes. Similar to the approach with GRA, we embedded stainless steel fiducials in the arms (one to two per arm) to visualize the integrity of the arms in the electronic device. The integrity of the electronics was observed directly using X-ray imaging. We observed that the integrated GRE exhibited an overall longer gastric residence period, which we hypothesize is due to the increase of size. The disintegration of the GRE was similar to that of the GRA. The two arms of the device detached on day 24 and 31 and the device passed out of the gastrointestinal tract within 41 d. We note that the maximum gastric residence period of GRE achieved was 36 d, out of three samples. Further studies in animals with higher gastric forces, such as dogs,^[38]^ can be performed for further characterization of gastric resident times of the devices.

The in vivo experiments with swine models demonstrate the ability of the prototype to sustain the mechanical stress in a large animal model. We note that variation of the gastric residence period between samples is likely due to inherent interanimal variation. In general, both GRA and GRE demonstrate a significant increase in gastric residence period in comparison to the electronics as shown in Figure 2G. The maximum period of gastric residence is 30 and 36 d, respectively, in comparison to a maximum residence of 3 d for the structure without the gastric residence architecture.

The ability to achieve a month-long residence with a three-armed gastric residence architecture enables the incorporation of active modules, which has not been previously demonstrated in gastric resident systems. For instance, as illustrated in Figure 1H, a wireless Bluetooth radio-frequency chipset, antenna, batteries, and drug delivery modules can be integrated into the device via a hybrid integration approach. The functioning electronics is readily capable of establishing wireless connection via a standard, widely adopted 2.4 GHz Bluetooth radio-frequency protocol. We first characterized the signal strength in vitro as shown in Figure 3A, where devices exhibited an average signal strength of −45 dBm at 30 cm, without power amplification (+0 dBm). We then assessed the signal strength while the device resided in the stomach, as shown in the in vivo experiments in Figure 3B. The distance was measured relative to the surface of the stomach of the pig in three directions to evaluate the directionality of the signal strength. The changes in signal strength at different angles in vivo are expected due to the asymmetric nature of organs. Nevertheless, despite the attenuation at 2.4 GHz transmission frequency caused by the tissue and fat surrounding the gastric cavity of a large animal (35–58 kg Yorkshire pig), we show the ability to maintain a stable interconnection with an off-the-shelf personal electronic device without additional hardware enhancement (electronics tablet and smart phone) (inset of Figure 3B). We demonstrated the ability to seamlessly interconnect with the user’s devices and simultaneously restrict the signal strength to within an arm’s length (e.g., −80 to −90 dBm at 45 cm). The limited connection range is a desirable security enhancement. The self-isolation of wireless signal strength within the user physical space could shield the device from unwanted connections, providing a physical isolation for additional security and privacy protection. Having validated the ability to receive advertised packets from the GRE, we then performed in vivo experiments to validate the ability to form bilateral Bluetooth connections with the GRE. This is demonstrated with a smart phone by directly connecting and requesting temperature measurement operations and receiving the temperature-sensing data from the GRE residing in the stomach of a Yorkshire pig. Figure 3C shows the increase in temperature from room temperature to core body temperature of a pig.

**Figure 3:**
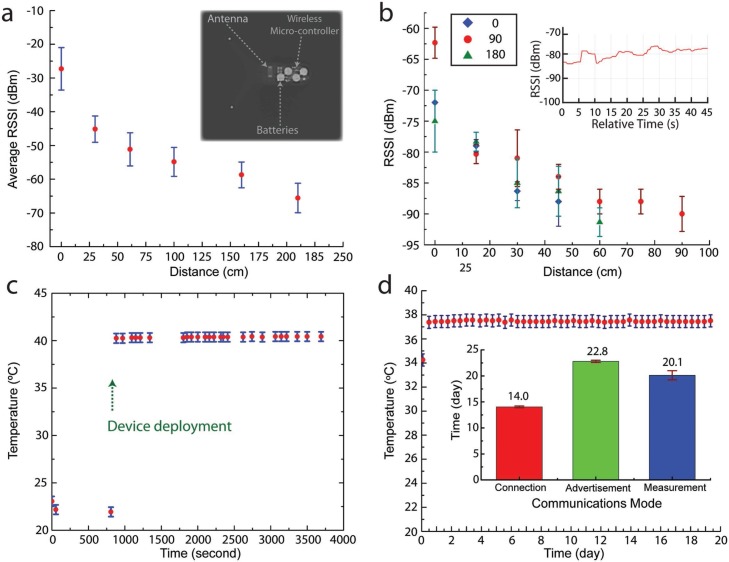
Wireless performance and lifetime of gastric-residence electronics. A) Average received signal strength indicator (RSSI) of seven devices measured with a smart phone. Inset shows an X-ray image of the integrated electronics at the GRE with three major components. B) The RSSI measured from GRE in a porcine stomach. The distance is measured relative to the abdominal surface of the pig. The in vivo measurements are repeated in three orthogonal directions. Inset shows the stability of RSSI measured at fixed location. C) GRE bilateral wireless communications from the gastric space: the change of temperature measured from GRE delivered to the porcine stomach, demonstrating the ability to perform bilateral wireless Blue-tooth interconnection between the device in the gastric space and a smart phone. D) Prolonging GRE lifetime: the optimization of communication protocol and power sources enable the maximum device lifetime of 20.1 d when configured to perform temperature measurement at an hourly interval. Inset shows bar charts demonstrate the average lifetime of GRE when maintained at three different modes of operation. The graph shows the in vitro experimental data of temperature measured when the device is left in a convection oven maintained at 37 °C over 19.5 d.

The integration of GRE with sensing elements could enable the creation of a long-term resident diagnostic platform. Having validated the ability to establish interconnection with the GRE in the pig, we focused on prolonging the device lifetime to achieve multi-week-long wireless functionalities. For example, we optimized the communication protocol to prolong the GRE communication lifespan. The goal is to maximize GRE lifetime by reducing power consumption without compromising the device’s ability to establish and maintain wireless interconnection with personal electronics. As shown in the inset of Figure 3D, we first compare the lifetime of GRE under Connection and Advertisement modes with a minimum functional communication frequency (Please refer to the Experimental Section for the details of the communication protocol). For instance, configuring the advertisement interval to 10 s enabled the GRE broadcasting lifetime to an average of 22.8 d under in vitro conditions. Further increases in advertisement interval beyond 10 s would result in challenges in establishing a stable Bluetooth connection. Conversely, a reduction of the interval would result in the decrease of device lifetime. Based on the experimental result, we designed an Android communication protocol to (1) seek the advertisement signal of a GRE based on the device unique identifier (media access control address, MAC address); (2) establish Bluetooth connection; (3) request temperature measurement to the GRE; (4) acquire and store temperature data in the Android platform; and (5) disconnect the GRE. This cycle is then repeated at a prescribed measurement interval. This enables the GRE to only establish connection as needed reducing overall power consumption by resuming to the low power advertisement mode until the next measurement point. As a proof of concept, we demonstrate in Figure 3D the ability to prolong the measurement to an average lifetime of 20.1 d for three GRE devices in an in vitro setting. A slight reduction in communication lifetime was observed when the device was configured to perform temperature measurement. This is expected due to the additional energy consumed in establishing connections, as shown in the Figure 3D inset.

The GRE architecture allows the integration of a drug delivery module, enabling the simultaneous controlled-release of drug during electronic sensing and operations. We demonstrate the ability to integrate a controlled release formulation of an antibiotic drug (doxycycline), as shown in Figure 4A. We tested drug release from this sustained release formulation of doxycycline, and compared it to immediate release and delayed release tablets. Drug release from the immediate release tablet in simulated gastric fluid was rapid. Almost all of the contents were released in less than 30 min. Drug release from the delayed release tablet lasted longer and a significant portion (≈80%) of the drug was released within 24 h. In contrast to tablets that are retained in the gastrointestinal tract for 1–2 d, the GRE is retained in the stomach for several weeks. Hence, drug release from the GRE is intended to be prolonged. To achieve this, we loaded doxycycline in a hydrophobic biodegradable matrix made of PCL. Drug release from the PCL matrix was gradual. After an initial burst of ≈10% in the first 0.5 h, drug was released at a near constant rate. 25 mg of the drug were released over one week. While this dosage is below the clinically efficacious dose, it serves as a proof of concept for sustained drug release using the GRE device.

**Figure 4:**
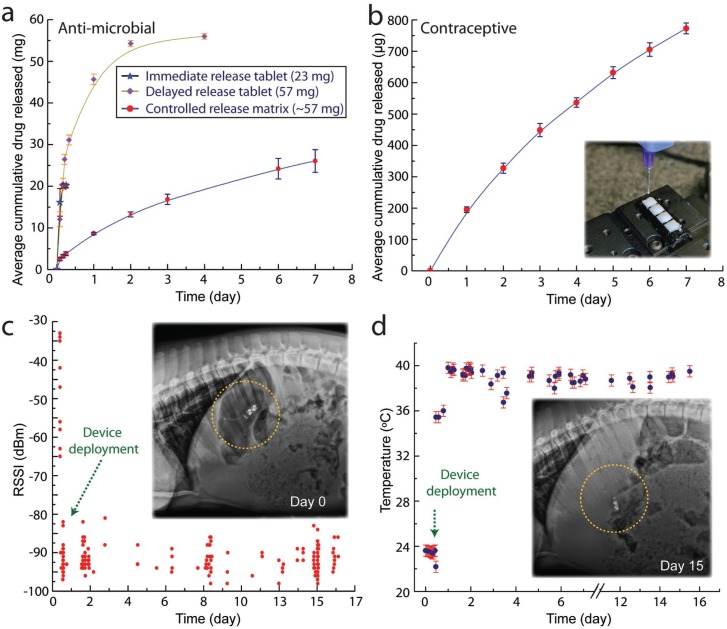
Drug delivery and remote sensing with gastric-residence electronics. A) The cumulative release of doxycycline in a controlled released poly(ε-caprolactone) matrix formulation (red dots and blue line), in comparison to the release profile of 20 mg in tablet form (blue stars and black line) and delayed released tablet (purple diamond and green line). B) The cumulative release of levonorgestrel from a 3D-printed formulation over one week, demonstrating the ability to integrate GRE with a controlled delivery platform. Inset shows the 3D printing of levonorgestrel into the defined drug wells. C) in vivo long-residence performance of GRE: The graph shows the measured received signal strength indicator (RSSI) of a GRE deployed in a porcine stomach of a pig from tablets attached to the walls of the cage over 15.3 d. Inset shows an X-ray image of the GRE (yellow circled) inside the stomach at the day the device is deployed. D) in vivo long-residence physiological parameter sensing with GRE: a direct, real-time core-temperature measurement with a tablet attached on the wall of the cage over 17 d. Inset shows the integrity of the GRE on day 15, demonstrating the robustness of the GRE to withstand the hostile gastric environment for weeks.

We also note that the entire drug-delivery device fabrication process is compatible with a desktop 3D printing process. As a proof of concept, we formulated a printable levonorgestrel releasing silicone matrix [poly(dimethylsiloxane)] (PDMS) that is 3D printed into the GRE structure (see the Experimental Section for a detailed description). We demonstrated that with the integration of a controlled-release formulation, levonorgestrel is released over a course of 6 d with an average of 106 μg d^−1^. Figure 4B shows the average cumulative drug release profile in simulated gastric fluid. Inset of Figure 4B shows the 3D printing of the formulated drug into 3D-printed drug wells. Such long-resident release of hormone could function as a long-resident hormone therapy platform, or to function as an ingestible contraceptive platform. Future work, which is beyond the focus of this study, can be performed to further optimize the release formulation to achieve desired pharmacokinetic performance in vivo.

The wireless connectivity of the GRE can be leveraged to incorporate various electrically activated modules. GRE is compatible with a wide range of actuation principles and can be designed based on the electrical power system affordable.

Indeed, such a platform can be designed to achieve electrically modulated drug delivery in addition to tailoring the polymeric matrix to achieve a range of drug release profiles demonstrated earlier. For example, we can achieve triggerable drug delivery with gold membranes^[8]^ which we have demonstrated previously.^[39]^ In another example, electrically activated modules through implementation of electroactive adhesive can potentially be developed to achieve drug delivery by microcompounding low melting temperature polymer with electrically conductive nanomaterials. (See the Supporting Information for a potential wirelessly triggerable mechanism for the cover release of a drug reservoir, Figure S4, Supporting Information, as well as the detail synthesis of the electroactive adhesive.) Unlike molding, 3D printing allows the cointegration of drugs formulated with distinct programmable release profile. This allows a seamless digital manufacturing methodology of long-resident drug delivery devices. We envision that such a rapid prototyping approach could enable the on-demand digitally defined creation of drug delivery GRE devices at a local healthcare facility by physicians and pharmacists, allowing next-generation personalized treatment strategies.

Indeed, the GRE is designed with a widely adapted Blue-tooth communication protocol and can interconnect with other clinical equipment, wearable or implantable sensors. The synergistic integration of electrically modulated drug release and device interconnection offers an exciting means of achieving digital-based biomedical diagnosis and intervention. We anticipate that the coupling of drug delivery modules with the advancement of on-body or implanted biosensors could ultimately enable a rapid, automated, or on-demand drug intervention to eliminate opportunistic infections prior to their growth and spreading as well as other applications where closed loop systems can help maximize the efficacy of an intervention on a clinical outcome.

As a proof of concept, we show multi-week-long physiological monitoring, such as core body temperature measurement, with a personal electronics compatible system via the GRE platform. This is achieved by integrating the three core advancements described earlier. First, the capability of the device to reside in the gastric space for a month (Figure 2). Second, the capability of establishing a direct, bilateral Bluetooth communications between commercially available personal electronics and the GRE residing inside the stomach of a large animal (Figure 3B,C). Third, the ability to prolong the GRE wireless electronics to weeks (Figure 3D). The integration of these core developments ultimately realize the demonstration of multi-week-long core-temperature measurement of a porcine pig model, as shown in Figure 4C,D. We note that the data gap recorded is due to the required physical clearance between the personal electronics (Android tablet) attached to the cage of the porcine model. This causes the GRE to be outside the range of connection (larger than 75 cm) when the porcine subject can move freely around the enclosure. Specifically, the Android tablets are attached at the wall of the cage with an area of 1.52 m × 1.52 m, and the tablet is placed 1.49 m above the ground. The distance is necessary to minimize the disruption of animals’ daily routine and to prevent the disruption of tablet operation. We note that we were successful in establishing connection with the GRE despite the distance, which falls at the weaker range (−100 to −80 dBm) as shown in Figure 3B. Such constraint will not be applicable in a human user where the personal electronics are used within the operational range.

The GRE demonstrated a 15.3 d operational lifetime inside the porcine stomach (Figure 4D). We note that the batteries were encapsulated within the device with a biocompatible polymer, PLA,^[40]^ to eliminate the potential risk of injury due to either the corrosive action or electrical burn upon contact with the GI tract.^[41]^ Further, unlike commonly used lithium ions based batteries with higher maximum current, the small (4.8 mm in diameter) silver oxide battery has a significantly lower likelihood of residence. In addition, it rapidly developed internal resistance during a short-circuit event, which self-limits its maximum output current in the unlikely incidence of battery contact with the GI tract. A single coin cell short circuit current is limited to a transient (orders of 4 s) spike of a short-circuited leakage current that is less than 22 mA. We note that the demonstrated lifetime is shorter than the gastric residence period as shown in Figure 2G, due to the limited energy capacity that can be accommodated by batteries in a limited size. We anticipate that with further advancement of integration, GRE can be powered by chemical energy harvested from gastric fluids,^[39]^ biodegradable batteries system,^[42]^ and wireless powering mechanism^[43]^ to safely prolong the device functionality in the hostile in vivo gastric environment.

In summary, the nonsurgical and needle-free transient implantation of wireless gastric resident electronic devices into the body has the capacity of providing a remote, direct diagnostic and therapeutic intervention. The ability to directly interface with portable consumer personal electronics such as smart phones, tablets and devices through a widely adopted wireless protocol empowers the users to directly communicate and control the long-residence gastric device without surgical procedures or other specialized equipment. This also enables a seamless interconnection with other wireless electronics peripherals, wearable devices, and biomedical implants, enabling a real-time feedback-based automated treatment or responsive medication. Indeed, the interconnection of ingestible gastric resident electronics with the digital cloud via personal electronics could enable remote health management and monitoring as well as data collection for clinical studies. Ultimately, the ingestible gastric residence electronics provides a needle and surgery free approach to synergistically integrate biomedical electronic devices, the human body, and the digital domain—realizing next-generation remote diagnostic and automated therapeutic strategies.

## Experimental Section

*GRA Fabrication*: 3D computer-aided design (CAD) models of GRA as shown in Figure 2A were first created with Solidworks 2016 (Dassault Systèmes). Stereolithography (STL) files were then digitally sliced and converted to print path in G-code (3D Slicer). The converted and optimized G-code were then 3D printed with a multimaterial FDM 3D Printer (System 30M, Hyrel 3D). PLA and thermoplastic polyurethane filaments (NinjaTek NinjaFlex 85A) with a diameter of 1.75 mm were used to create the stiff and elastomeric components, respectively. The 3D-printed GRA were embedded with stainless steel imaging probes (1 mm beads) to enable X-ray visualization of the printed device inside the gastric cavity (see inset of Figure 2A).

*Control Device Fabrication*: Control device (head) of Figure 2C was 3D printed with the same procedure as GRA (described previously) but with the gastric residence architecture (arms) removed. The converted and optimized G-code were then 3D printed with a multimaterial FDM 3D Printer (System 30M, Hyrel 3D). PLA with a diameter of 1.75 mm was used to create the structure. Every control device was then embedded with three stainless steel imaging probes in a row (1 mm beads), spaced ≈4 mm apart, to enable X-ray visualization of the printed device inside the gastric cavity (see inset of Figure 2C).

*GRE Fabrication*: GRE was 3D printed with the same procedure as GRA (described previously) but with a CAD model integrated with electronics and batteries as shown in Figure 1H. A 2.4 GHz Bluetooth wireless electronics board (Texas Instruments) and coin cell batteries were assembled and integrated with the 3D-printed GRA structure. Epoxy (3M United States) and conductive traces were 3D printed with a custom-built 3D printer (AGS 15000, Aerotech Inc.). The 3D printer dispensing was modulated with a digital pneumatic regulator (Ultimus V High Precision Dispenser, EFD). Localized heating was applied to the 3D-printed PLA with a solder iron to seal remaining gap of the 3D-printed parts.

*In Vivo Experiments*: All procedures were conducted in accordance with the protocols approved by the Massachusetts Institute of Technology Committee on Animal Care. In vivo porcine studies were performed in female Yorkshire pigs aged between four and eight months and weighing ≈35–58 kg. In vivo experiments were not blinded or randomized. Prior to endoscopy or administration of the prototypes the animals were fasted overnight immediately prior to the procedure. On the day of the procedure for the endoscopic characterization and deployment studies the animals were anesthetized with intramuscular injections of Telazol (tiletamine/zolazepam, 5 mg kg^−1^), xylazine (2 mg kg^−1^), and atropine (0.04 mg kg^−1^), the pigs were intubated and maintained on inhaled isoflurane (1–3%). The esophagus was intubated with an esophageal overtube (US Endoscopy). The prototypes were delivered directly to the stomach through the overtube using the endoscope to pass the prototypes. Animals were radiographed periodically to assess prototype location. A total of ten stomach-deposited devices were evaluated in ten separate pig experiments (three for control, four for GRA, and three for GRE), see Figure 2G. Animals were monitored twice daily for changes in fecal output, abdominal distension, lethargy, inappetence, and any signs of discomfort. There were no abnormal clinical findings in any of the animals dosed with the device.

*Wireless Performance*: To characterize the wireless electronics performance, the RSSI strength of seven devices were first measured in ambient conditions within the range of 210 cm with a smart phone. To characterize the RSSI strength of the device In vivo, the device was delivered to the stomach of a Yorkshire pig (51 kg) as described in the procedure earlier. The RSSI was measured with an Android tablet relative to the abdominal surface of the pig at three orthogonal directions. Similarly, the device was delivered to the stomach of a pig as described earlier to evaluate the performance of bilateral communications by performing temperature measurement In vivo (Figure 3C).

*Lifetime Optimization and Characterization*: To characterize the lifetime of GRE, the devices were configured to communication protocol (advertisement, connection) and to perform temperature measurement requested by an Android platform (Android 5.0, Google). The maximum operational lifetimes were determined from the collected packets for advertisement and connection experiments. Specifically, for the connection test, GRE was configured to maintain Bluetooth connection at a distance of 1.2 m from the central device with the following connection parameters: “Connection Interval” of 240 ms, a “Slave latency” setting of 49. For the advertisement test, GRE was configured to advertise at 10 s interval and the packets were measured accordingly to determine the lifetime. For temperature measurement test, a customized Android program was built (Android Studio, Google) to seek and establish connection based on the specified MAC address of the GRE, initiate temperature measurement command at GRE, transmit and store temperature data, disconnect the device before repeating the cycle at a prescribed measurement interval. Long-term in vitro temperature measurements (Figure 3D) were performed in an incubator at 37 °C at 100 RPM with a measurement interval of 1 h. The maximum operational lifetime was calculated from the time-point of the collected temperature readings.

*Doxycycline Controlled Release Formulation*: To prepare sustained release formulations of doxycycline, doxycycline hyclate (20% by weight) and poly(ε-caprolactone) (37 kDa) (80% by weight) were weighed in a glass vial. The glass vial was then placed in a convection oven and heated to 90 °C to melt the polymer. Once the polymer melted, the contents of the vial were mixed vigorously to evenly distribute the drug powder. The mixture was placed in the oven again, and upon melting was transferred into drug reservoirs. The drug reservoirs were weighed before filling the formulation and after filling to obtain the amount of drug loaded.

*In Vitro Drug Release of Doxycycline*: To analyze drug release from the sustained release doxycycline formulation, the formulations synthesized above were placed in 25 mL simulated gastric fluid (SGF) in a shaker incubator 37 °C and 100 RPM. At various times, a part of the release medium was aliquoted and frozen to −20 °C until further characterization. The rest of the release media was discarded and replaced with fresh media. The study was carried out for one week. On completion of the study, drug concentration in the release media aliquoted at various times was determined using high-performance liquid chromatography (HPLC). HPLC was performed on an Agilent 1260 Infinity HPLC system. Chromatographic separation was carried out on an AdvanceBio RP-mAb SB-C8 column (4.6 × 100 mm, 3.5 m particle size) placed at 55 °C. The mobile phase consisted of a mixture of 20 × 10^−3^ m potassium phosphate buffer (pH 6) (60%) and acetonitrile (40%). The mobile phase was flown at 0.85 mL min^−1^ for an HPLC run time of 4 min. For each analysis 5 μL sample was injected onto the column, and UV absorbance was monitored at λmax = 350 nm.

*3D Printable Levonorgestrel Controlled Release Formulation*: The levonorgestrel release formulation combined 30% of levonorgestrel (Astatech) with 70% of PDMS (Dow Corning) by mass in a homogeneous viscous suspension. The mixture was printed into the 3D-printed drug reservoirs and solidified in a convection oven overnight.

*In Vitro Drug Release of Levonorgestrel*: Drug release from 3D-printed formulations of levonorgestrel was tested in 25 mL of SGF in a shaker incubator at 37 °C and 100 RPM. The remaining steps of the analysis were performed by a method identical to the one described for doxycycline. For HPLC analysis of levonorgestrel, a Poroshell 120, EC-C18, 4.6 × 50 mm column with 2.7 μm particle size was used. The column was maintained at 50 °C. A gradient method was developed and the mobile phase consisted of water and acetonitrile. The mobile phase started as 100% aqueous at time zero, and was changed linearly to 100% organic phase over 2 min. The composition was held at 100% organic phase for the next 2.5 min, and then changed back 100% aqueous phase over the next 0.1 min. This gave a total run time of 4.6 min, followed by a post-time of 1.25 min. Detection was carried out at 250 nm.

*In Vivo Long-Term Temperature Monitoring*: In vivo experiments were conducted according to the procedure described earlier (see In vivo Experiments section). For long-term temperature monitoring, the pig was left to move around freely in the enclosure with an area of 1.52 m × 1.52 m. Android tablets were placed 1.49 m above the ground to prevent disruption. The device was configured to advertise at 8 s advertisement interval. Two tablets were configured to collect advertised packets to assess the electronics lifetime of the device (Figure 4C). An additional tablet was dedicated to establish bilateral connection with GRE to measure core temperature at a minimum interval of an hour (Figure 4D).

## Supporting Information

Supporting Information is available from the Wiley Online Library or from the author.

## References

[1] Kim D. H., Lu N., Ma R., Kim Y. S., Kim R. H., Wang S., Wu J., Won S. M., Tao H., Islam A., Yu K. J., Kim T. I., Chowdhury R., Ying M., Xu L., Li M., Chung H. J., Keum H., McCormick M., Liu P., Zhang Y. W., Omenetto F. G., Huang Y., Coleman T., Rogers J. A., *Science* 2011, 333, 838. 21836009 10.1126/science.1206157

[2] Gao W., Emaminejad S., Nyein H. Y. Y., Challa S., Chen K., Peck A., Fahad H. M., Ota H., Shiraki H., Kiriya D., Lien D. H., Brooks G. A., Davis R. W., Javey A., *Nature* 2016, 529, 509. 26819044 10.1038/nature16521 PMC4996079

[3] Nathan D. A., Center S., Wu C.-Y., Keller W., *Am. J. Cardiol.* 1963, 11, 362. 13937691 10.1016/0002-9149(63)90130-9

[4] Sarna S., Bowes K., Daniel E., *Gastroenterology* 1976, 70, 226. 1248682

[5] Benabid A. L., Chabardes S., Mitrofanis J., Pollak P., *Lancet Neurol.* 2009, 8 67. 19081516 10.1016/S1474-4422(08)70291-6

[6] Silva J. N. A., Bromberg B. I., Emge F. K., Bowman T. M., Van Hare G. F., *J. Am. Heart Assoc.* 2016, 5. 10.1161/JAHA.116.003632 PMC4937287 27231019

[7] Evans D. F., Pye G., Bramley R., Clark A. G., Dyson T. J., Hardcastle J. D., *Gut* 1988, 29, 1035. 3410329 10.1136/gut.29.8.1035 PMC1433896

[8] Santini J. T., Cima M. J., Langer R., *Nature* 1999, 397, 335. 9988626 10.1038/16898

[9] Farra R., Sheppard N. F., McCabe L., Neer R. M., Anderson J. M., Santini J. T., Cima M. J., Langer R., *Sci. Transl. Med.* 2012, 4, 122ra21. 10.1126/scitranslmed.3003276 22344516

[10] a) Lee S. H., Lee Y. B., Kim B. H., Lee C., Cho Y. M., Kim S.-N., Park C. G., Cho Y.-C., Choy Y. B., *Nat. Commun.* 2017, 815032;

[11] a) Veiseh O., Doloff J. C., Ma M., Vegas A. J., Tam H. H., Bader A. R., Li J., Langan E., Wyckoff J., Loo W. S., Jhunjhunwala S., Chiu A., Siebert S., Tang K., Hollister-Lock J., Aresta-Dasilva S., Bochenek M., Mendoza-Elias J., Wang Y., Qi M., Lavin D. M., Chen M., Dholakia N., Thakrar R., Lacik I., Weir G. C., Oberholzer J., Greiner D. L., Langer R., Anderson D. G., *Nat. Mater.* 2015, 14, 643; 25985456 10.1038/nmat4290 PMC4477281

[12] Anderson J. M., *Cardiovasc. Pathol.* 1993, 2, 33.

[13] Bevan J., *The Simon and Schustler Handbook of Anatomy and Physiology*, Simon & Schuster, New York, 1978.

[14] a) Mintchev M. P., Deneva M. G., Aminkov B. I., Fattouche M., Yadid-Pecht O., Bray R. C., *Physiol. Meas.* 2010, 31, 131; 20009188 10.1088/0967-3334/31/2/001

[15] DeBakey M., Ochsner A., *Surgery* 1939, 5, 132.

[16] Gorter R. R., Kneepkens C. M. F., Mattens E. C. J. L., Aronson D. C., Heij H. A., *Pediatr. Surg. Int.* 2010, 26, 457. 20213124 10.1007/s00383-010-2570-0 PMC2856853

[17] a) Imaz I., Martínez-Cervell C., García-Álvarez E. E., Sendra-Gutiérrez J. M., González-Enríquez J., *Obes. Surg.* 2008, 18, 841; 18459025 10.1007/s11695-007-9331-8

[18] a) Gordi T., Hou E., Kasichayanula S., Berner B., *Clin. Ther.* 2008, 30, 909; 18555937 10.1016/j.clinthera.2008.05.008

[19] Bellinger A. M., Jafari M., Grant T. M., Zhang S., Slater H. C., Wenger E. A., Mo S., Lee Y. L., Mazdiyasni H., Kogan L., Barman R., Cleveland C., Booth L., Bensel T., Minahan D., Hurowitz H. M., Tai T., Daily J., Nikolic B., Wood L., Eckhoff P. A., Langer R., Traverso G., *Sci. Transl. Med.* 2016, 8, 365ra157. 10.1126/scitranslmed.aag2374 PMC5264553 27856796

[20] Kirtane A. R., Abouzid O., Minahan D., Bensel T., Hill A. L., Selinger C., Bershteyn A., Craig M., Mo S. S., Mazdiyasni H., Cleveland C., Rogner J., Lee Y. L., Booth L., Javid F., Wu S. J., Grant T., Bellinger A. M., Nikolic B., Hayward A., Wood L., Eckhoff P. A., Nowak M. A., Langer R., Traverso G., *Nat. Commun.* 2018, 9, 2. 29317618 10.1038/s41467-017-02294-6 PMC5760734

[21] Zworykin V., Farrar J., *Nature* 1957, 179, 898.

[22] a) Bettinger C. J., *Trends Biotechnol.* 2015, 33, 575; 26403162 10.1016/j.tibtech.2015.07.008

[23] Lesho J. C., Hogrefe A. F., 1989.

[24] a) Cassilly D., Kantor S., Knight L. C., Maurer A. H., Fisher R. S., Semler J., Parkman H. P., *Neurogastroenterol. Motil.* 2008, 20, 311; 18194154 10.1111/j.1365-2982.2007.01061.x

[25] Watson B. W., Ross B., Kay A. W., *Gut* 1962, 3, 181. 14005272 10.1136/gut.3.2.181 PMC1413324

[26] Mimee M., Nadeau P., Hayward A., Carim S., Flanagan S., Jerger L., Collins J., McDonnell S., Swartwout R., Citorik R. J., Bulović V., Langer R., Traverso G., Chandrakasan A. P., Lu T. K., *Science* 2018, 360, 915. 29798884 10.1126/science.aas9315 PMC6430580

[27] a) Schaefer G., *US5792048A*, 1998;

[28] Kalantar-Zadeh K., Berean K. J., Ha N., Chrimes A. F., Xu K., Grando D., Ou J. Z., Pillai N., Campbell J. L., Brkljača R., *Nat. Electron.* 2018, 1, 79.

[29] a) Iddan G., Meron G., Glukhovsky A., Swain P., *Nature* 2000, 405, 417; 10.1038/35013140 10839527

[30] a) Gross J., *US5318557A*, 1994;

[31] Salessiotis N., *Am. J. Surg.* 1972, 124, 331. 5056893 10.1016/0002-9610(72)90036-0

[32] Haller M., Ferek-Petric B., Donders A. P., *US7181505B2*, 2004.

[33] Schork N. J., *Nature* 2015, 520, 609. 25925459 10.1038/520609a

[34] Ghosh U., Ning S., Wang Y., Kong Y. L., *Adv. Healthcare Mater.* 2018, 7, 1800417. 10.1002/adhm.201800417 30004185

[35] a) Aoyagi N., Ogata H., Kaniwa N., Uchiyama M., Yasuda Y., Tanioka Y., *J. Pharm. Sci.* 1992, 81, 1170; 1491333 10.1002/jps.2600811208

[36] a) Kirschenbaum M. D., Safaya A., Pee S., *MIS Case Rep. SLS* 2017;

[37] Zhang S., Bellinger A. M., Glettig D. L., Barman R., Lee Y.-A. L., Zhu J., Cleveland C., Montgomery V. A., Gu L., Nash L. D., Maitland D. J., Langer R., Traverso G., *Nat. Mater.* 2015, 14, 1065. 26213897 10.1038/nmat4355 PMC4772966

[38] Laulicht B., Tripathi A., Schlageter V., Kucera P., Mathiowitz E., *Proc. Natl. Acad. Sci. USA* 2010, 107, 8201. 20404209 10.1073/pnas.1002292107 PMC2889561

[39] Nadeau P., El-Damak D., Glettig D., Kong Y. L., Mo S., Cleveland C., Booth L., Roxhed N., Langer R., Chandrakasan A. P., Traverso G., *Nat. Biomed. Eng.* 2017, 1, 0022. 28458955 10.1038/s41551-016-0022 PMC5404703

[40] Uhrich K. E., Cannizzaro S. M., Langer R. S., Shakesheff K. M., *Chem. Rev.* 1999, 99, 3181. 11749514 10.1021/cr940351u

[41] Laulicht B., Traverso G., Deshpande V., Langer R., Karp J. M., *Proc. Natl. Acad. Sci. USA* 2014, 111, 16490. 25368176 10.1073/pnas.1418423111 PMC4246317

[42] Bettinger C. J., Whitacre J., *US9985320B2*, 2015.

[43] a) Abid A., O’Brien J. M., Bensel T., Cleveland C., Booth L., Smith B. R., Langer R., Traverso G., *Sci. Rep.* 2017, 7, 46745; 28447624 10.1038/srep46745 PMC5406829

