# Supplementary material for: 3D-Printed Gastric Resident Electronics
Source: Adv Mater Technol. 2018 Dec 13;4(3):1800490. doi: 10.1002/admt.201800490 (PMC6988123; doi:10.1002/admt.201800490)
Supplement: Supplementary file 1 [file ADMT-2019-201800490-S1.pdf]

Copyright WILEY-VCH Verlag GmbH & Co. KGaA, 69469 Weinheim, Germany, 2018.

ADVANCED MATERIALS TECHNOLOGIES

Supporting Information

for *Adv. Mater. Technol.,* DOI: 10.1002/admt.201800490

3D-Printed Gastric Resident Electronics

Yong Lin Kong, Xingyu Zou, Caitlin A. McCandler, Ameya R. Kirtane, Shen Ning, Jianlin Zhou, Abubakar Abid, Mousa Jafari, Jaimie Rogner, Daniel Minahan, Joy E. Collins, Shane McDonnell, Cody Cleveland, Taylor Bensel, Siid Tamang, Graham Arrick, Alla Gimbel, Tiffany Hua, Udayan Ghosh, Vance Soares, Nancy Wang, Aniket Wahane, Alison Hayward, Shiyi Zhang, Brian R. Smith, Robert Langer, and Giovanni Traverso**

Supporting Information

Title 3D Printed Gastric Resident Electronics

Author(s), and Corresponding Author(s)*
Yong Lin Kong, Xingyu Zou, Caitlin A. McCandler, Ameya R. Kirtane, Shen Ning, Jianlin Zhou, Abubakar Abid, Mousa Jafari, Jaimie Rogner, Daniel Minahan, Joy E. Collins, Shane McDonnell, Cody Cleveland, Taylor Bensel, Siid Tamang, Graham Arrick, Alla Gimbel, Tiffany Hua, Udayan Ghosh, Vance Soares, Nancy Wang, Aniket Wahane, Alison Hayward, Shiyi Zhang, Brian R. Smith, Robert Langer*, Giovanni Traverso*

GRA folding force measurement:
Funnel test apparatus was used to simulate the passage of GRA through the pylorus, where the experiment was set up as described with the prior publications by Bellinger et al.[19] and Kirtane et al.[20] (See Figure S3C of [19] and Supplementary Figure 2 of [20] for detailed information of the set-up). In these experiments, the GRA prototype was pushed by an aluminum rod in a mechanical tester (Instron) to a maximum displacement of 13 mm and the maximum folding force were measured. As described in Figure S1, the folding force evaluated with GRA ranges from an average of 7.7 N (at the first cycle) to 7.0 N (after 10000 cycles). This folding force from a three-arms architecture is larger than prior folding force measurement of 3.5 N for a six-arm gastric residence devices developed by Bellinger et al.[19] (See Supporting Information Figure S3C of [19]). GRA also maintained the folding forces after 10000 cycles, with a relatively smaller degree of reduction of folding force (10%) in comparison to the result shown by Kirtane et al.[20]

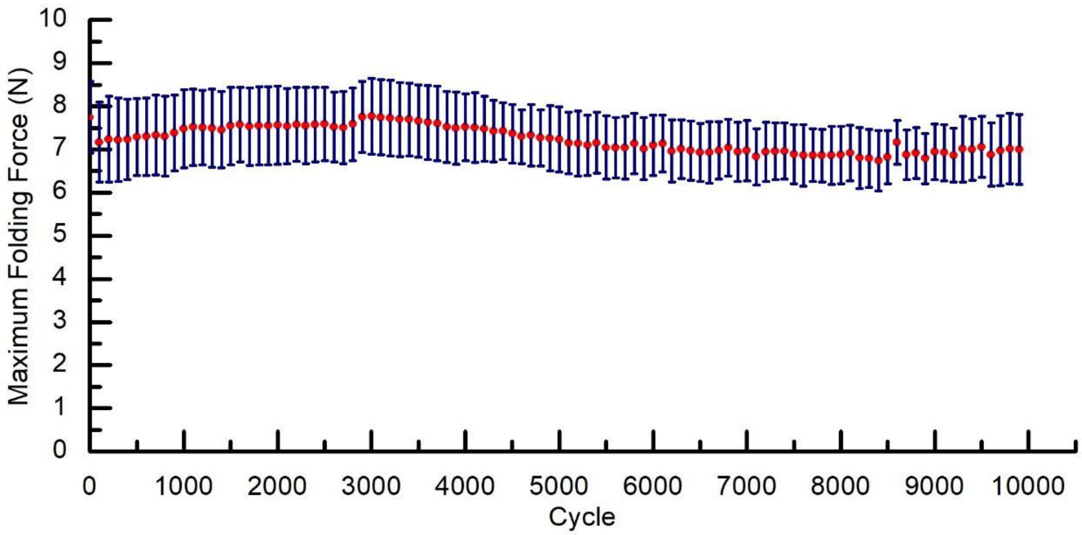

Figure S1: Maximum folding force measured with funnel test apparatus to simulate the passage of GRA through pylorus. The measurement was repeated for 10000 cycles to evaluate the fatigue properties. Note that only 1 out of 100 points is plotted in this graph to clearly illustrate the standard deviation.

PCL-PLA GRA: We demonstrate the ability to incorporate PCL-based polyurethane by first co-printing PLA with water-soluble polyvinyl alcohol (PVA) as a supporting structure. 3D

Computer Aided Design (CAD) models of GRA as shown in Figure 2A were first created with Solidworks 2016 (Dassault Systèmes) as described previously, with the exception of modifying of co-printing with PVA instead of thermoplastic polyurethane (orange region of the Figure 2A). Stereolithography (STL) files were then digitally sliced and converted to print path in G-code (3D Slicer). The converted and optimized G-code were then 3D printed with a multi-material Fused Deposition Modeling (FDM) 3D Printer (Ultimaker 3, Ultimaker). PLA and PVA filaments (Ultimaker) with a diameter of 2.85 mm were used to create the stiff and supporting components respectively where the PVA is then removed with water. PCL elastomer is synthesized by first mixing of a 6:1.3:0.027:9.5 molar ratio of PCL diol (MW, 530, Sigma Aldrich), PCL triol (MW, 900, Sigma Aldrich), linear PCL (MW, 45,000, Sigma Aldrich), and hexamethylene diisocyanate (Sigma Aldrich) as described in [19]. The prepolymer is then casted into the removed PVA structure (orange region of the Figure 2A) of the 3D printed model rested on a negative mold to create the PCL-PLA based GRA structure. The PCL-PLA GRA structure demonstrates a folding force from an average of 6.7 N (at the first cycle) to 6.5 N (after 3000 cycles), as described in Figure S2. This is in the same order as the folding force described for GRA at Figure S1. The folding force decreases from 6.5 N after 3000 cycles to 3.1 N after 5980 cycles, which is due to the weakening and subsequently a fracture of one of the gastric residence arm. This is likely to be due to crack propagated from the microscopic bubble in the casted PCL elastomer. Future work can improve the materials synthesis process to improve the fatigue resistance of the device, for instance by developing a PCL elastomer 3D printing strategy to replace the casting process. In summary, we show that the fabrication procedure of GRA and GRE can be modified to incorporate thermoset plastic that cannot be directly 3D printed through FDM. This, for instance, can potentially enable the incorporation of FDA-approved materials and novel responsive material such as enteric polymers that can further minimize potential clinical complications.

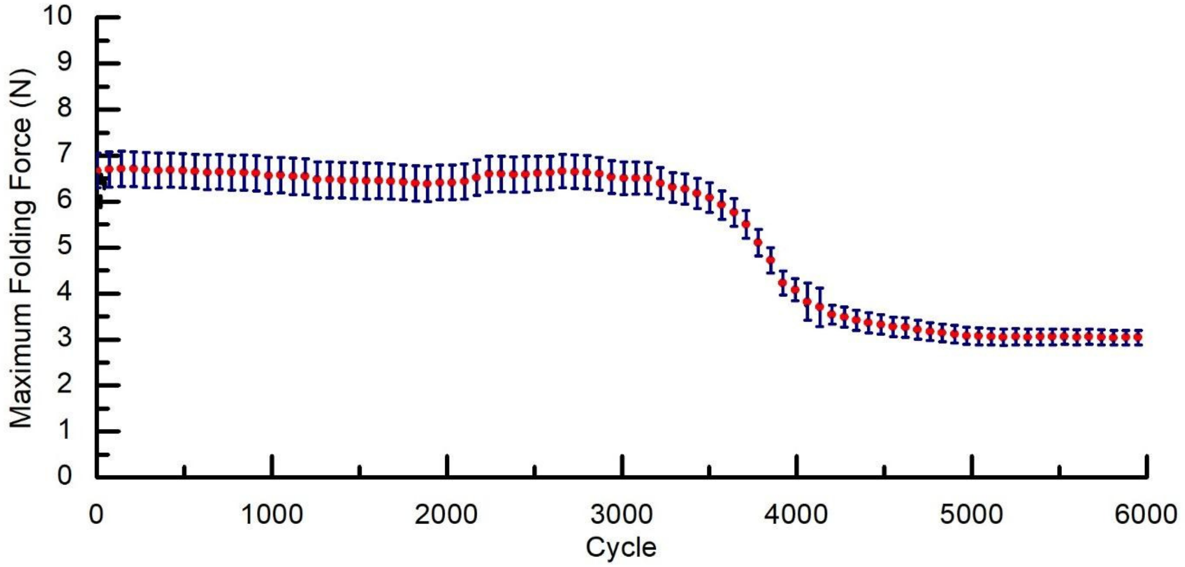

Figure S2: Maximum folding force measured with funnel test apparatus to simulate the passage of PCL-PLA GRA through pylorus. The measurement was repeated for 6000 cycles to evaluate the fatigue properties. Note that only 1 out of 70 points is plotted in this graph to clearly illustrate the standard deviation.

Remote triggering demonstrations: Two proof-of-concept experiments were performed in vivo to demonstrate the ability to achieve remote triggering with GRE in the stomach of a large animal

model. Specifically, electroactive adhesive was developed to achieve GRA separation from electronics as well as remote-delivery of drugs.

Synthesis of electroactive adhesive: First, a low melting temperature electrically conductive nanocomposite was synthesized. Specifically, a poly(ε-caprolactone) (Sigma Aldrich) and 10 wt% carbon nanotubes (Sigma Aldrich) are mixed with twin-screw micro-compounding (Xplore™ Instruments, Netherlands) to create a 3D printable filament with an average diameter of 1.75 mm and an electrical conductivity of 100 Sm^{-1}. The electroactive adhesive was electrically connected to a microcontroller switch in the GRE via printed conductive traces. The electroactive adhesive was used to compress a spring with the 3D printed PLA structures. Upon wireless triggering with Android tablet, Joule heating would melt the composite matrix to weaken the adhesive strength, allowing the stored elastic energy in the spring to cause structural separation. We show that such triggering can be achieved in vivo in the gastric cavity, as shown in the following endoscopy image sequences. We anticipate that future work, which is beyond the scope of this paper, will include a long-term in vivo assessment on the robustness of this interface and optimization of power consumption to reduce the device footprint.

(1) In vivo triggered GRA separation: To demonstrate the ability to achieve device separation, a GRA was bonded with electroactive adhesive (see "*Synthesis of Electroactive adhesive"* above for the detail synthesis of the electroactive adhesive) to a "head" of 3D printed GRE. The device was delivered to the stomach of a pig (see "In vivo experiments" at Experiment Methods for detail description of in vivo procedure.) To help the capturing of the separation process, the GRA arms were tied. As describe in Figure S3A, the device was initially intact. The separation is then triggered via an Android tablet where the GRA was separated after a minute. A slight movement of the separated structure shows that GRA was completed detached from the "head" of the GRE. Figure S3C shows the separated device where the compression system (an embedded spring) can be observed.

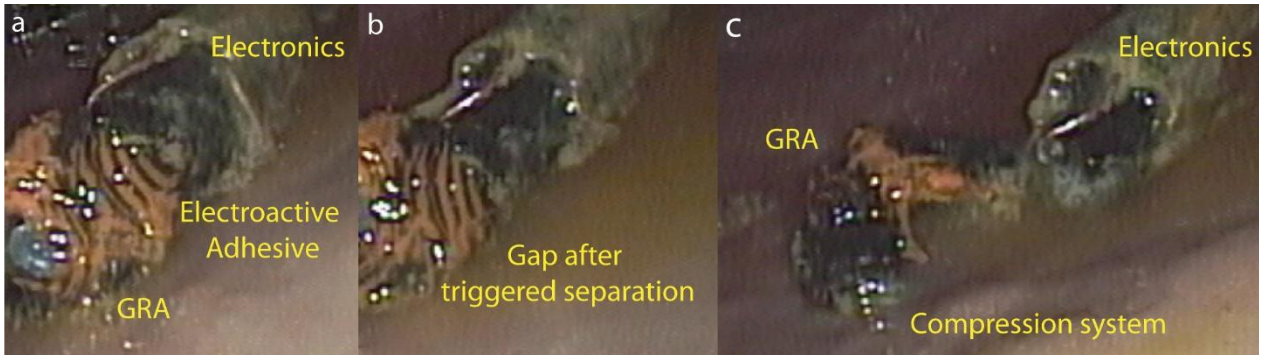

Figure S3: Triggerable GRE device separation. (**A**) Prior to triggering, the GRA is bonded to the "head" of GRE electronics with electroactive adhesive. **(B)** Upon triggering, the GRA is separated as the adhesive failed. **(C)** A slight movement of the separated structure shows that GRA was completed detached from the "head" of the GRE where the compression system (an embedded spring) of the device was exposed.

(2) In vivo wireless triggerable release of drug-reservoir cover: Gold, which is otherwise inert in acidic environment, can be electrochemically corroded by shifting the electrochemical potential. We have previously shown that using gold as the drug release membrane, ingestible electronics

can be used to power the release of micro-gram of model drug (methylene blue) in a reservoir (2 mm × 1 mm × 1.5 mm) that is sealed with a 300 nm thick gold membrane. During corrosion, the maximum power consumption required is 0.8 mW, which is well within the maximum power affordable by the GRE system (45 mW). To demonstrate the ability to achieve wireless large volume drug delivery, a 3D printed PLA drug window (4 mm × 4 mm × 0.5 mm) encapsulate the doxycycline powder reservoir 3D printed at the "head" of 3D printed GRE where the electroactive adhesive compressed a spring (see "*Synthesis of Electroactive adhesive"* above for detail synthesis of the electroactive adhesive). As shown in Figure S4A, the device was delivered to the stomach of a pig with procedure as described earlier (see "In vivo experiments" at Experiment Methods). Upon triggering, the Joule heating of the electroactive adhesive causes the release of the reservoir cover, allowing the infiltration of gastric fluid to dissolve the encapsulated drugs as shown in Figure S4B. We note that the triggered opening of the reservoir cover was successful despite of the mucosal coverage on the delivery site. (See the attached Supplementary Videos). Figure S4C shows the compression system after the mucous covering the triggered well was removed by injecting water through endoscope. This experiment was repeated with two-different pigs with two other devices and were all successful. We hypothesize that the infiltration of gastric fluid into the opened drug cover will dissolve the water-soluble doxycycline. Here, we have demonstrated the ability to achieve the wireless release of drug-reservoir cover. Such system should be compatible to store ingestible pills for delivery. We note that further detail in vivo experiments with pharmacokinetic studies are needed to fully demonstrate the efficacy of drug delivery for the specific drug of interest.

In summary, we demonstrate that we are able to achieve on-demand mechanical and structural changes with the GRE chipset, which can be used for releasing drug-containing reservoir in vivo and other potential applications.

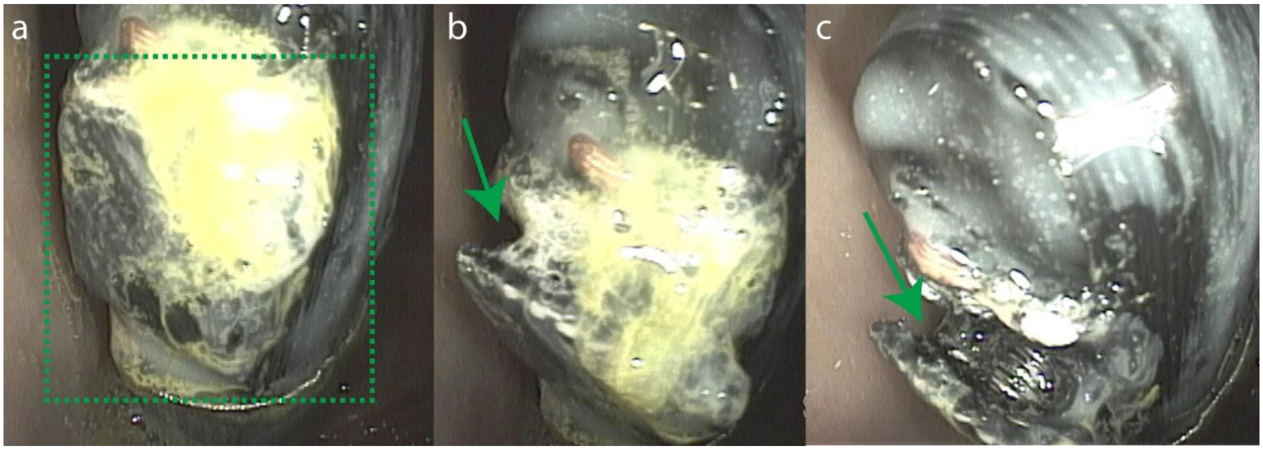

Figure S4: In vivo wireless triggerable release of drug-reservoir cover (A) Endoscopy images show the electractive drug delivery module (green dashed-line box) prior to triggering. Mucous films from the stomach covers reservoir. **(B)** The wireless triggered release of drug as a result of the openning of drug reservoir cover (green arrow) which was not interfered by the mucous coverage. **(C)** Washed triggered reservoir to show the expanded system (green arrow).

Supplementary movies S1, S2: Supporting videos show the in vivo triggerable release of drug-reservoir covers of two representative devices in a porcine stomach.
